# Mutations Within the Transcription Factor *PROP1* in a Cohort of Turkish Patients with Combined Pituitary Hormone Deficiency

**DOI:** 10.4274/jcrpe.galenos.2020.2019.0191

**Published:** 2020-09-02

**Authors:** Fatma Derya Bulut, Semine Özdemir Dilek, Damla Kotan, Eda Mengen, Fatih Gürbüz, Bilgin Yüksel

**Affiliations:** 1 Adana City Training and Research Hospital, Clinic of Pediatrics, Adana, Turkey; 2 Çukurova University Faculty of Medicine, Department of Pediatrics, Division of Pediatric Endocrinology, Adana, Turkey; 3 Ankara City Hospital, Children’s Hospital, Clinic of Pediatric Endocrinology, Ankara, Turkey

**Keywords:** Combined pituitary hormone deficiency, hypopituitarism, pituitary transcription factors, PROP1 gene

## Abstract

**Objective:**

Mutations of the genes encoding transcription factors which play important roles in pituitary morphogenesis, differentiation and maturation may lead to combined pituitary hormone deficiency (CPHD). *PROP1* gene mutations are reported as the most frequent genetic aetiology of CHPD. The aim of this study was to describe the phenotypes of Turkish CPHD patients and define the frequency of *PROP1* mutations.

**Methods:**

Fifty-seven CPHD patients from 50 families were screened for *PROP1* mutations. The patients were affected by growth hormone (GH) and additional anterior pituitary hormone deficiencies.

**Results:**

All patients had GH deficiency. In addition, 98.2% had central hypothyroidism, 45.6% had hypogonadotropic hypogonadism, 43.8% had adrenocorticotropic hormone deficiency and 7.1% had prolactin deficiency. Parental consanguinity rate was 50.9% and 14 cases were familial. Mean height standard deviation score (SDS) and weight SDS were -3.8±1.4 and -3.1±2.0, respectively. Of 53 patients with available pituitary imaging, 32 (60.4%) showed abnormalities. None had extra-pituitary abnormalities. Eight index patients had *PROP1* gene mutations. Five sporadic patients were homozygous for c.301_302delAG (p.Leu102CysfsTer8) mutation, two siblings had exon 2 deletion, two siblings had complete gene deletion and two siblings were homozygous for the novel c.353A>G (p.Q118R) mutation. The frequency of the *PROP1* mutations was 16% in our cohort. Mutation rate was significantly higher in familial cases compared to sporadic cases (42.8% vs 11.6%; p<0.01).

**Conclusion:**

Phenotype of patients regarding hormonal deficiencies, pituitary morphology, presence of extra-pituitary findings, family history of CPHD and parental consanguinity are important for deciding which pituitary transcription factor deficiency should be investigated. *PROP1* mutation frequencies vary in different populations and its prevalence is high in Turkish CPHD patients.

**What is already known on this topic?**

The *PROP1* gene product is a critical transcription factor for development and maintenance of proper functioning of the anterior pituitary gland. To date, *PROP1* gene mutations are reported to be the most frequent genetic aetiology of combined pituitary hormone deficiency (CPHD) and these mutations are associated with progressive anterior pituitary hormone deficiencies.

**What this study adds?**

The frequency of *PROP1* gene mutations in a Turkish cohort of CPHD patients is reported. Pathogenic mutations were detected in 11 of 57 (19.3%) patients and gross deletions were present. A novel variant was discovered in two siblings. Clinical patient characteristics and treatment responses are also described.

## Introduction

Combined pituitary hormone deficiency (CPHD) is defined as deficiencies of growth hormone (GH), thyroid-stimulating hormone (TSH), the gonadotropins-luteinizing hormone (LH) and follicle-stimulating hormone (FSH), prolactin (PRL) and adrenocorticotropic hormone (ACTH). Worldwide prevalence of CPHD is estimated as 1/8000 (1).

Both in human and mice, pituitary organogenesis and maintenance of its proper functioning necessitates the appropriate expression of a cascade of signalling molecules and transcription factors which are crucial for organ commitment, cell proliferation, patterning and terminal differentiation (2,3,4).

The genes that are related to these transcription factors are *PROP1, POU1F1, (PIT1), LHX3, LHX4*, and *HESX1*. In 1998, Wu et al (5) identified homozygous or compound heterozygous inactivating mutations of *PROP1* gene as being associated with CPHD. To date, *PROP1* mutations are the most commonly reported genetic aetiology of CPHD in humans (4,5,6). Prophet of PIT-1 *(PROP1)* is a paired-like homeobox 1 gene, located on chromosome 5q35.3 and consists of three exons encoding for a 226-amino acid protein which is a late-expressed transcription factor (4). Mutations of *PROP1* gene cause autosomal recessively inherited CPHD and clinical phenotypes include GH, TSH, FSH/LH, PRL and rarely ACTH deficiencies together with morphological pituitary anomalies (4,7).

Phenotypes associated with *PROP1* gene mutations can be highly variable. Deficiencies of all pituitary hormones may be present with varying severity and at different ages. However, in all cases anterior pituitary function deteriorates over time (4,8). TSH and GH deficiencies have a tendency to occur in early childhood, whereas gonadotropin and corticotropin deficiencies manifest later in life (4,8). As *PROP1* is a “later-acting transcription factor”, extra-pituitary manifestations are not observed (8). Magnetic resonance imaging (MRI) of the anterior pituitary gland shows normal or enlarged gland in early stages and pituitary involution in later stages whilst size and location of the posterior pituitary is normal and pituitary stalk interruption is not observed (1,4). Rarely, pituitary masses associated with *PROP1* gene mutations are reported (4,9,10). Point mutations, small and large deletions and insertions in the *PROP1* gene have been reported but there are no associations between any specific variants and specific regions or ethnicities (11,12,13).

The aim of this study was to define patient characteristics and to identify *PROP1* gene mutations in our CHPD patient cohort.

## Methods

### Study Design and Patient Selection

This retrospective cohort study was conducted in Çukurova University Research and Education Hospital including 57 patients with combined anterior pituitary hormone deficiency who attended the hospital between January 1997 and August 2019. Exclusion criteria for the patients were isolated GH deficiency (GHD), brain tumour, central nervous system surgery, cranial-neck irradiation, systemic chronic illnesses or chromosomal abnormalities.

Patients who were diagnosed with CPHD were analysed for *PROP1* mutations. The patients included in the study were all affected by GHD and at least one additional anterior pituitary hormone deficiencies including TSH, gonadotropins, ACTH or PRL. Diagnosis was based on clinical, laboratory and imaging investigations. Serum GH, insulin-like growth factor-1 (IGF-1), IGF-binding protein-3 and plasma ACTH concentrations were analysed by commercial kits using Siemens immulite 2000 immunoassay system and FSH, LH, oestradiol, cortisol, TSH, free thyroxine and PRL were analysed by commercial kits using Beckman Coulter Unicel Dxl 800 immunoassay system based on electrochemiluminescence immunoassay.

Genomic DNA was isolated from peripheral leucocytes. *PROP1* gene (transcript ID: ENST00000308304.2 and protein ID: O75360) was screened by polymerase chain reaction (PCR) amplifications of exons and neighbouring intronic regions. The PCR products were purified and directly sequenced using the Big Dye terminator cycle sequencing ready reaction kit (PE Applied Biosystems, Foster City, Calif., USA) in an ABI PRISM 3130 automatic sequencer (PE Applied Biosystems, Foster City, Calif., USA). DNA sequence data analyses were evaluated with DNA Sequencing Analysis Software-Sequencher 5.0 programme (http://genecodes.com/). All of the variants were investigated using 1000 genomes browser database (http://browser.1000genomes.org/index.html) and the National Center for Biotechnology Information database (https://www.ncbi.nlm.nih.gov/clinvar) as to whether they were novel or previously reported. Subsequently, mutant variants were interpreted by *in silico* prediction tools such as Mutation Taster, SIFT and PolyPhen-2 (14,15,16).

Segregation analysis was performed only for the family of patients with the novel variant. It was not possible to test the parents of the patients with known pathogenic variants due to financial limitations.

PCR amplification of certain exons of the *PROP1* gene failed for initial DNA samples obtained from nine patients. Four patients from two families gave consent for further testing and multiplex ligation dependent probe amplification (MLPA) assays were performed only for these patients. The other five patients were not included in the calculation of mutation frequency.

Patients from the same family are indicated with the same superscript letter.

The Ethics Committee of the Çukurova University Faculty of Medicine approved this study (approval: #TF2013LTP24), and written informed consent was obtained for each patient or from their legal guardians.

### Statistical Analysis

Data obtained from this study were analysed using SPSS statistical software, version 23.0 (IBM Inc., Armonk, NY, USA). The distribution of data was evaluated with the Kolmogorov-Smirnov test. For numerical comparisons, the independent sample t-test or Mann-Whitney U tests were used for parametric and non-parametric distribution of the measured parameters, as appropriate. Descriptive statistics which were not normally distributed were presented as median and range. Frequency distributions and percentages were given for categorical variables.

## Results

All 57 of the patients included to the study were affected by GHD and diagnosed in childhood. In addition, 56 patients (98.2%) had central hypothyroidism, 26 (45.6%) had hypogonadotropic hypogonadism, 25 (43.8%) had ACTH deficiency and four (7.1%) had PRL deficiency. More than two-thirds of the patients were male (68.4%). Median age age at diagnosis was 7.7 years (range: 3 months-19.8 years). Mean delay in bone age at diagnosis was 3.3±2.4 years. More than half of the patients (n=29; 50.9%) had parental consanguinity and 14 patients were familial cases. There was no history of perinatal asphyxia or difficult birth. None of the patients had any major dysmorphic findings. Height standard deviation score (SDS) at diagnosis was -3.8±1.4. Weight SDS at diagnosis was -3.1±2.0. IGF-1 SDS at diagnosis was -3.0±1.5). Median age at the start of GH replacement treatment was 8.5 years (range: 3 months - 20 years). All of the patients received appropriate treatments for their hormonal deficiencies. Twelve patients achieved their final height and mean final height SDS for these cases was -1.0±0.7. Final height and target height values for *PROP1* mutated patients are shown in Table 1.

Pituitary MRI was available for 53 patients. Twenty-one had normal pituitary MRI, 17 had pituitary hypoplasia, eight had hypoplasia of the adenohypophysis, three had ectopic neurohypophysis and three had pituitary adenoma. Patient 22 had pituitary adenoma, which resolved on follow-up and had transformed into anterior pituitary hypoplasia. None had extra-pituitary abnormalities on MRI.

Patients 14, 22, 41, 46 and 57 had homozygous deletion of c.301_302delAG in exon 2 of the *PROP1* gene. This mutation resulted in frame-shift and premature stop codon (p.Leu102CysfsTer8). These five patients had different combinations of anterior pituitary hormone deficiencies. All had GH and TSH deficiencies at the time of diagnosis. Four of these patients, who have reached the age of puberty, showed clinical and laboratory findings consistent with hypogonadotropic hypogonadism. Only one (patient 22) had ACTH deficiency and none had PRL deficiency (Table 1). Patients 14, 22, 41 and 46 responded quite well to the GH and levothyroxine supplementations and appropriate hormone replacement to induce secondary sex characteristics. Individual responses of the patients to GH replacement are shown in Table 1. Patient 57 was newly diagnosed and was recently started on GH replacement.

PCR amplification of second and third exons of *PROP1* gene had failed for DNA of patients 1^a^-2^a^, 7^d^-8^d^, 9^e^-10^e^ and 15 all of whom had parental consanguinity. No pathogenic mutations were detected within exon 1 for these patients. MLPA assay was only performed for patients 1^a^ and 2^a^ from the same family and a homozygous deletion of exon 2 of the *PROP1* gene were detected in both siblings. These two brothers have GHD at the time of diagnosis and developed TSH deficiency after approximately one or two years. Both had delayed pubertal development and lack of secondary male sex characteristics due to hypogonadotropic hypogonadism. Eventually, both developed ACTH deficiency (Table 1).

PCR amplification of the whole *PROP1* gene had failed for DNA from patients 58^f^-59^f^. MLPA assays detected complete gene deletion in these siblings. The elder sister showed GHD at the age of two-and-a-half years and developed TSH deficiency four years later. When she reached the age of puberty, she developed both ACTH deficiency and hypogonadotropic hypogonadism. Her younger brother showed both TSH and GH deficiencies at diagnosis; he is currently prepubertal and is not affected by ACTH insufficiency (Table 1). Pituitary imaging revealed pituitary adenoma in patient 58^f^ but was normal in 59^f^. Adenoma did not exhibit progression and remained stable.

**Figure 1:**
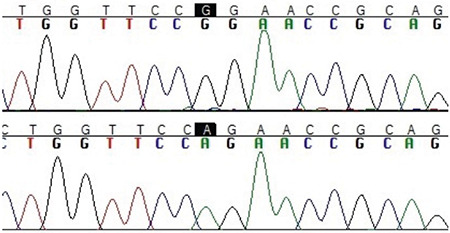
Sequencing electropherogram of patients 3^b^ and 4^b^

Patients 3^b^ and 4^b^ from the same family with the same phenotype had homozygous c.353A>G (p.Q118R) variant in exon 3 of the *PROP1* gene (Figure 1). This novel variant was predicted to be disease-causing by *in silico* predictive tools such as Mutation Taster, SIFT and PolyPhen-2 due to splice site changes and possibly affected protein features (14,15,16). Both parents, who were consanguineous, and a healthy sister of the patients were heterozygous for the same mutation. Both siblings had GHD at the time of diagnosis and a few years later they developed TSH deficiency. They showed hypogonadotropic hypogonadism and PRL deficiency in adolescence (Table 1). On physical examination, decreased body hair growth and pubic hair growth were marked in both siblings. Patient 4^b^ had pituitary adenoma on pituitary MRI. On follow-up, she had visual impairment and consequently underwent pituitary surgery.

## Discussion

In this study, *PROP1* gene mutations were detected in eight index patients from a cohort of 57 CPHD patients from 50 families. Segregation analysis of the variants in the pedigrees revealed three patients with the same pathogenic *PROP1* mutations. More than half of the patients with mutation were familial cases and positive mutation frequency was significantly higher in familial cases compared to sporadic cases (3/7 familial cases versus 5/43 sporadic cases, p<0.01).

There are several reports of cohorts defining genetic aetiology of CPHD from different parts of the world. *PROP1* gene mutations are reported to be the most frequent amongst both sporadic and familial CPHD patients (4,6,8,17). However, the frequency was reported to vary widely between 0% and 70.1% from different populations (10,18,19,20,21). *PROP1* mutation frequencies among CPHD patients are highest in Eastern European populations especially Lithuanian, Polish and Hungarian, and also high in Portuguese, Russian and Brazilian cohorts (3,10,12,22,23,24,25,26,27,28). In contrast, *PROP1* mutation rates are usually low in Western and Southern European countries, Australia and in cases with Asian origin, especially in sporadic CPHD patients (3,6,18,19,20,21,29). *PROP1* gene mutations are not rare among Turkish CPHD patients (13,30). In 2014, Baş et al (30) screened 76 Turkish CPHD patients and the frequency of *PROP1* mutations was 21.8%. *PROP1* mutation frequency in this study was similar to our study. Kandemir et al (13) reported *PROP1* mutations in another Turkish cohort which was present in two familial patients while 51 sporadic CPHD patients were mutation negative. In our study, we detected *PROP1* mutations in 16% patients. Interestingly, Kandemir et al (13) detected lower *PROP1* mutation prevalence compared to our study. This might be attributed to dissimilarities in ethnicity, parental consanguinity rate and frequency of familial cases between these three Turkish cohorts. Overall evaluation of Turkish CPHD patients from previous studies together with the patients from our study gives an estimated frequency of *PROP1* gene mutations of 16.6% amongst Turkish CPHD patients. In addition to their study, De Rienzo et al (6) reviewed all CPHD cases retrospectively and postulated that *PROP1* gene mutations are responsible for 11.2% of all CPHD cases.

*PROP1* mutation prevalence is higher in familial patients compared to sporadic cases in all cohorts (3,6,13,22,24,26,29,30,31,32). Parental consanguinity is known to increase the risk for autosomal recessive conditions. Thus, parental consanguinity would appear to be a risk factor for *PROP1* mutations (12,22,30). This hypothesis is supported by evidence from our study, with an overall parental consanguinity rate of 50.9% which increased to 81.8% amongst *PROP1* mutated patients. If the cases are sporadic, that is that there is a single affected individual in a family, and there is no parental consanguinity, the aetiology is more likely to be acquired rather than genetic (1,3,4).

The c.301_302delAG mutation was reported to be one of the most prevalent mutations of *PROP1* gene (2,8,10,11,26). This mutation is a two base pair deletion which results in a frameshift and early termination of the protein at codon 109. Dusatkova et al (2) investigated this variant and suggested that the reason for the high occurrence rate may be a founder effect rather than a variant hot spot (2). This assumption was made by haplotype analyses and the geographic distribution of the c.301_302delAG variant which was interpreted as suggesting an ancestral origin. Five of our patients had this variant and exhibited variable hormone deficiencies. Large deletions were detected in four patients. Many studies, in which CPHD patients from different populations including Turkish patients were screened for *PROP1* deficiency, reported homozygous deletions of the entire gene or particular exons (7,30,33). For this reason, MLPA analysis should be a routine part of genetic investigation in MPHD patients.

The previously unreported p.Q118R substitution is interpreted as likely pathogenic considering the concordance of phenotype, parental consanguinity and segregation analyses of the variant. This variant is anticipated to be important as it is highly conserved in different orthologues. In addition, it is located in the homeobox domain (5). In 1998, Wu et al (5), identified p.F117I and p.R120C substitutions and they postulated that these variants allowed protein binding but with reduced affinity. As, p.Q118R variant is present in the sequence between these variants, it is assumed that this variant is also associated with pathogenicity due to altered protein function. In silico analyses with Mutation Taster, SIFT and PolyPhen-2 also indicate likely alteration of protein features and splice site changes (14,15,16).

Patients with *PROP1* mutations typically have clinical manifestations of GHD in early childhood. TSH and PRL deficiencies often coexist at the time of diagnosis. At the onset of puberty, patients usually do not exhibit secondary sexual characteristics due to hypogonadotropic hypogonadism. Rarely, some patients show pubertal changes and hypogonadotropic hypogonadism may develop later in adulthood. ACTH deficiency occurs variably as the patient grows older (1,4). As a result, these patients should be carefully monitored for occurrence of other anterior pituitary hormone deficiencies. It is postulated that this phenomenon of progressive hormone deficiency is due to dysfunction of *PROP1* in initiating pituitary stem cell migration and differentiation (34). Patients with *PROP1* mutations lack extra-pituitary manifestations (6,8,31). All of the *PROP1* mutated patients in our cohort had GH and TSH deficiency at the time of diagnosis in early childhood. Nine patients had hypogonadotropic hypogonadism when puberty should have been evident and the other two patients were prepubertal. The two siblings with the novel mutation had remarkably sparse body and pubic hair. ACTH deficiency was observed in half of the patients and the patients without ACTH deficiency are continuing to be monitored as usually ACTH deficiency is the last hormonal deficiency to occur, if it does. Onset age of progressive hormonal deficiencies differ in patients with the same mutations and even in familial cases in our cohort. A clear phenotype-genotype correlation has not been proposed in the literature, since progressive hormonal deficiencies occur at different chronologies even in individuals with the same genotype (4,17,24).

Response to GH treatment was satisfactory in our patient cohort and was similar to previous reports (32). Final height was achieved in nine of the *PROP1* mutated patients, all of whom had final height SDS in the mid-parental target height SDS range. This result was in agreement with previous reports (10,35,36).

MRI of the hypophysis commonly reveals pituitary hypoplasia or aplasia in these patients but occasionally pituitary hyperplasia evolving to hypoplasia and pituitary masses have been reported. (1,4,6,37,38). In contrast, ectopic posterior lobe and stalk abnormalities have not been observed (4). Interestingly, anterior pituitary MRI was normal in six patients. Three more patients had adenoma, two had hypoplasia and one initially had adenoma which evolved into pituitary hypoplasia. Pituitary morphology can change during follow-up of patients with *PROP1* gene mutation (9). None of our patients showed extra-pituitary manifestations on neuroimaging. Patients with adenoma have different genotypes; two had the common homozygous c.301_302delAG mutation, one had a novel mutation and one had complete gene deletion. Of interest, two of these cases were familial, and their siblings had normal pituitary gland upon MRI. With the exact genetic aetiology, patients with pituitary adenoma have the opportunity to avoid unnecessary invasive procedures (1).

### Study Limitations

Five of the patients with failed PCR amplification were not available for further testing with MLPA analysis. There is a high probability that a large deletion may exist in the *PROP1* gene of these familial CPHD cases with parental consanguinity which would have increased the proportion of MPHD patients with *PROP1* mutations in our cohort. In this study, we were not able to test the parents of all patients with pathogenic mutations due to financial limitations. For future studies, patients without any mutations identified in the *PROP1* gene may be screened for the other genes of pituitary transcription factors and gene panels may be more cost-effective for this purpose.

## Conclusion

It is crucial to screen GHD patients regularly for other anterior pituitary hormone deficiencies. With the exact genetic aetiology, the family is able to receive genetic counselling, unnecessary laboratory testing can be avoided and at the same time the opportunity of predicting the typical phenotype and developing hormonal deficiencies can be detected earlier. If the patients are familial and have parental consanguinity, genetic testing would be even more cost-effective.

## Figures and Tables

**Table 1:**
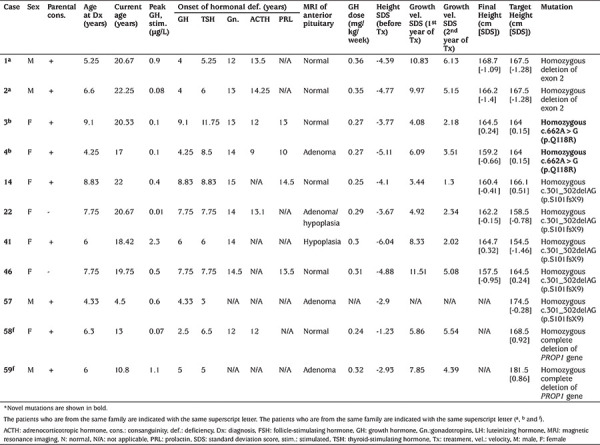
Clinical features and genotype of the patients with PROP1 gene mutations
